# Effect of Family and Personal Medical History on Treatment Outcomes of Tyrosine Kinase Inhibitors (TKIs) in Non-Small Cell Lung Cancer (NSCLC)

**DOI:** 10.3390/healthcare13151810

**Published:** 2025-07-25

**Authors:** Heves Surmeli, Ezgi Turkoglu, Deniz Isik, Oguzcan Kinikoglu, Yunus Emre Altintas, Ugur Ozkerim, Sila Oksuz, Tugba Basoglu, Hatice Odabas, Nedim Turan

**Affiliations:** Department of Medical Oncology, Kartal Dr. Lütfi Kirdar City Hospital, Health Science University, Istanbul 34865, Türkiye; ezgiturk_90@hotmail.com (E.T.); dnz.1984@yahoo.com (D.I.); ogokinikoglu@yahoo.com (O.K.); yunusaltintas1688@gmail.com (Y.E.A.); ugur.ozkerim@hotmail.com (U.O.); sila.oksuz@gmail.com (S.O.); basoglutugba@gmail.com (T.B.); odabashatice@yahoo.com (H.O.); turan.nedim@hotmail.com (N.T.)

**Keywords:** non-small cell lung cancer, tyrosine kinase inhibitors, family medical history, treatment outcomes, recurrence

## Abstract

**Background**: Tyrosine kinase inhibitors (TKIs) have significantly improved outcomes in non-small cell lung cancer (NSCLC), especially among patients with actionable genetic mutations. However, the influence of family and personal medical history (FPMH) on clinical and treatment outcomes with TKI therapy remains underexplored. **Methods**: We conducted a retrospective cohort study involving 136 NSCLC patients receiving TKIs, categorized into two groups based on the presence or absence of documented FPMH. Clinical variables assessed included demographic data, comorbidities, Eastern Cooperative Oncology Group (ECOG) performance status, tumor characteristics, genetic mutations (EGFR, ALK, ROS1), treatment responses, toxicity profiles, and survival outcomes. Statistical analyses included Chi-square tests, *t*-tests, Mann–Whitney U tests, Spearman correlation, and univariate logistic regression (*p* < 0.05 threshold for significance). **Results**: Patients with FPMH (n = 34) had a significantly higher burden of chronic diseases (58.8% vs. 15.7%), poorer ECOG scores (≥3: 8.8% vs. 1.0%), increased recurrence (41.2% vs. 20.6%), and greater chemotherapy-related toxicity (50.0% vs. 28.4%) compared to those without FPMH (n = 102). However, there were no significant differences in survival duration or mutation status between the two groups. **Conclusions**: FPMH may be a predictive factor for treatment complications and recurrence in NSCLC patients receiving TKIs, although it does not appear to influence survival or genetic mutation status. These findings support the need for personalized clinical monitoring strategies based on medical history.

## 1. Introduction

Lung cancer remains the leading cause of cancer-related mortality globally, with non-small cell lung cancer (NSCLC) accounting for approximately 85% of all cases. NSCLC includes several histologic subtypes such as adenocarcinoma, squamous cell carcinoma, and large cell carcinoma. Despite advances in screening and treatment, most NSCLC cases are diagnosed at an advanced stage, and the overall 5-year survival remains below 25% [1,2].

A variety of risk factors contribute to NSCLC pathogenesis, including smoking, environmental exposures (e.g., radon, asbestos), and genetic predisposition [3,4,5]. In recent years, targeted therapies such as tyrosine kinase inhibitors (TKIs) have become essential for NSCLC patients with actionable mutations, particularly in EGFR, ALK, and ROS1 genes [6,7,8]. These therapies have substantially improved outcomes, particularly in patients with adenocarcinoma and non-smoking status. However, there remains heterogeneity in treatment response, even among patients with similar genetic mutations.

One underexplored factor potentially influencing treatment outcomes is family and personal medical history (FPMH). FPMH refers to a documented history of malignancy or chronic disease in the patient or first-degree relatives. Previous studies have shown that family history of cancer may correlate with a higher prevalence of actionable mutations and may also reflect inherited cancer susceptibility [9,10]. Similarly, a patient’s own medical history, including prior malignancy or chronic comorbidities (e.g., diabetes, cardiovascular disease), may impact drug metabolism, immune response, and tolerance to therapy [11,12,13].

Despite its clinical relevance, few studies have evaluated the role of FPMH in NSCLC patients undergoing TKI therapy. One retrospective analysis by Obiols et al. (2022) suggested that a family history of cancer might influence survival outcomes with TKIs, but the sample size was limited, and conclusions were inconclusive [14]. Moreover, existing literature often overlooks the impact of comorbidities, functional status, and recurrence risk in this context.

Therefore, this study aims to systematically evaluate the effect of FPMH on clinical and treatment outcomes in NSCLC patients treated with TKIs. Specifically, it investigates differences in comorbidity burden, ECOG performance status, recurrence, toxicity, survival, and genetic biomarkers between patients with and without FPMH. Understanding whether FPMH influences TKI treatment response may support more individualized approaches in NSCLC management.

## 2. Materials and Methods

### 2.1. Study Design

This was a retrospective cohort study designed to assess the effect of family and personal medical history (FPMH) on clinical and treatment outcomes in patients with non-small cell lung cancer (NSCLC) treated with tyrosine kinase inhibitors (TKIs). Specifically, we aimed to (1) compare clinicopathological characteristics between patients with and without FPMH, (2) evaluate the prognostic relevance of clinical variables including FPMH using survival analysis, and (3) identify variables associated with the presence of FPMH.

### 2.2. Definition of FPMH

FPMH was operationally defined as the presence of either of the following:

Family history: A first-degree relative (parent, sibling, or child) diagnosed with cancer or a major chronic illness such as cardiovascular disease, diabetes, or hypertension.

Personal history: Documented diagnosis of chronic diseases (e.g., diabetes mellitus, chronic obstructive pulmonary disease, hypertension) or a prior cancer diagnosis in the patient themselves.

We acknowledge that some chronic diseases included in the FPMH definition may be potential risk factors for NSCLC or influence treatment outcomes (e.g., affecting ECOG performance or drug metabolism). To address this, additional statistical adjustments and subgroup analyses were performed.

### 2.3. Patient Population

We reviewed medical records of 162 NSCLC patients treated with TKIs at Kartal Dr. Lütfi Kirdar City Hospital from January 2020 to June 2023. After excluding 26 patients due to missing essential data, the final sample included 136 patients, stratified into two groups:

**FPMH group:** 34 patients.

**Non-FPMH group:** 102 patients.

Inclusion criteria:

Histologically confirmed NSCLC.

Received at least one line of TKI therapy.

Complete medical records including clinical history, treatment response, and follow-up.

### 2.4. Data Collection

We extracted the following variables:

Demographic: Age, sex, smoking status, and pack-years.

Clinical: ECOG performance status, comorbidities, FPMH status, and recurrence.

Pathological: Tumor histology, TNM stage (AJCC 8th edition), and PD-L1 expression.

Molecular: EGFR, ALK, and ROS1 mutation status.

Treatment: Type and duration of TKI, chemotherapy, toxicity, and surgical intervention.

Outcome: Overall survival (OS), progression-free survival (PFS), toxicity, and recurrence.

### 2.5. Statistical Analysis

Descriptive statistics were presented using means ± standard deviations for continuous variables and frequencies (%) for categorical variables.

Group comparisons used Chi-square/Fisher’s exact tests (categorical), *t*-tests (parametric continuous), or Mann–Whitney U tests (non-parametric).

Correlation analysis: Spearman’s rank correlation coefficient (non-parametric) was used to assess associations between FPMH and clinical variables.

Survival analysis:

Kaplan–Meier (K–M) curves for OS and PFS were plotted and compared using the log-rank test.

Cox proportional hazards regression was applied to estimate hazard ratios (HR) and 95% confidence intervals for key predictors (age, ECOG, stage, FPMH, mutation status).

Predictor analysis of FPMH:

A logistic regression model was built with FPMH as the dependent variable.

Independent predictors included comorbidity, ECOG score, recurrence, and molecular status.

A *p*-value < 0.05 was considered statistically significant. Statistical analyses were performed using SPSS v25 (IBM Corp., Armonk, NY, USA).

## 3. Results

A total of 136 NSCLC patients treated with TKIs were analyzed, including 102 patients without a family and personal medical history (FPMH) and 34 patients with FPMH. The comparison between groups is summarized below.

### 3.1. Demographic and Clinical Characteristics

The gender distribution and smoking status were similar between groups. Female representation was 54.9% (56/102) in the non-FPMH group and 47.1% (16/34) in the FPMH group (*p* = 0.552). Non-smokers made up 62.7% (64/102) of the non-FPMH group and 55.9% (19/34) of the FPMH group (*p* = 0.612).

However, patients with FPMH had a significantly higher prevalence of chronic diseases (58.8% vs. 15.7%, *p* < 0.001) and poorer performance status as measured by the Eastern Cooperative Oncology Group (ECOG) score (≥3 in 8.8% vs. 1.0%, *p* = 0.048). ECOG scores reflect patients’ functional status and range from 0 (fully active) to 5 (deceased).

**Figure 1:**
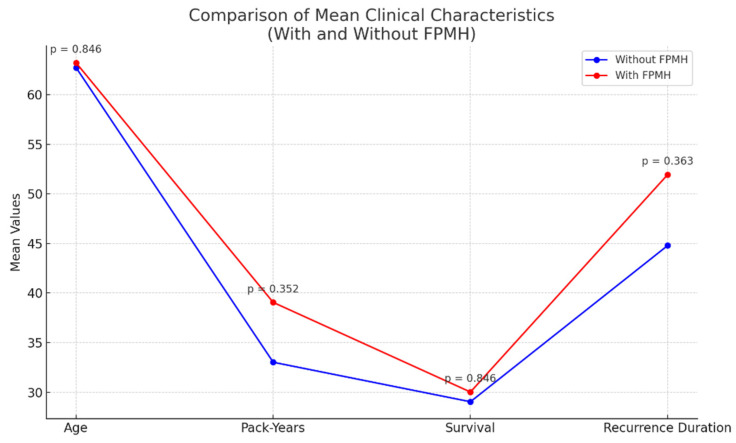
Comparison of mean clinical characteristics between family and personal medical history (FPMH).

As shown in Table 1, patients with a family or personal medical history (FPMH) of disease had a significantly higher prevalence of chronic disease (58.8% vs. 15.7%, *p* < 0.001) and recurrence post-treatment (41.2% vs. 20.6%, *p* = 0.031) compared to those without FPMH. Also, as shown in Figure 1, patients with a family or personal medical history (FPMH) of disease exhibited higher mean values for their pack-years, recurrence duration, and chronic disease prevalence, although the differences in their age and survival were not statistically significant.

### 3.2. Tumor Characteristics, Staging, and Genetic Markers

TNM staging and molecular profiles were largely similar across both groups. TNM classification, which assesses tumor size, lymph node involvement, and metastasis, showed no statistical difference (early-stage [I–II]: 17.6% non-FPMH vs. 23.5% FPMH; *p* = 0.720) [14].

**Figure 2:**
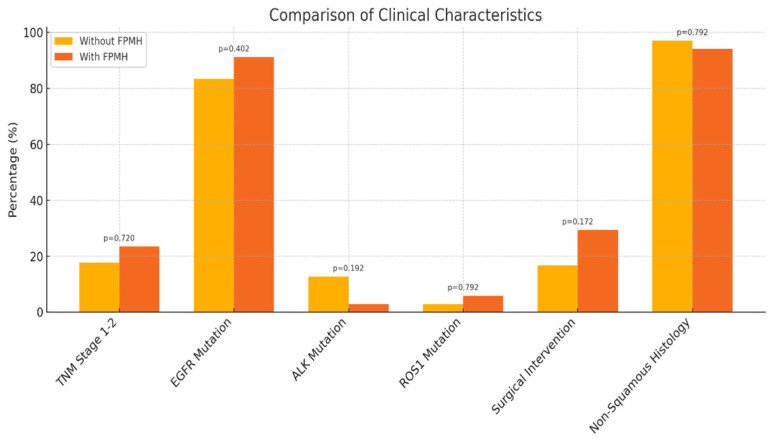
Comparison of clinical characteristics by FPMH status in patients (n = 136).

Genetic mutations, including EGFR, ALK, and ROS1, also did not differ significantly. EGFR mutations were present in 83.3% of non-FPMH patients and 91.2% of FPMH patients (*p* = 0.402). Similarly, surgical rates and histological subtypes showed no significant difference. Figure 2 highlights that tumor stage and genetic biomarkers such as EGFR, ALK, and ROS1 mutations were not significantly different between groups [14,15,16].

As presented in Table 2, no statistically significant differences were observed between the FPMH and non-FPMH groups in tumor stage, EGFR mutation status, or other genetic biomarkers.

### 3.3. Treatment Characteristics and Toxicity

Chemotherapy-related toxicity was significantly more common among patients with FPMH (50.0% vs. 28.4%, *p* = 0.036), while rates of neoadjuvant therapy, adjuvant chemotherapy, and metastatic status were similar (*p* > 0.05 for all). The duration of metastatic chemotherapy and response rates were also statistically equivalent [17].

**Figure 3:**
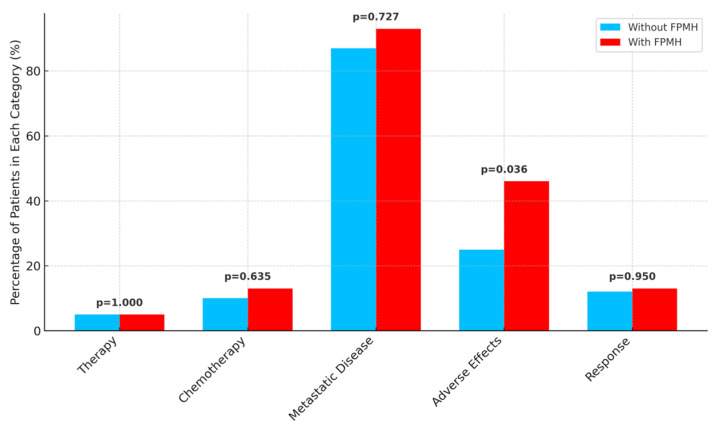
A bar chart showing therapy rates, metastatic status, adverse effects, and response rates, with *p*-values labeled.

**Figure 4:**
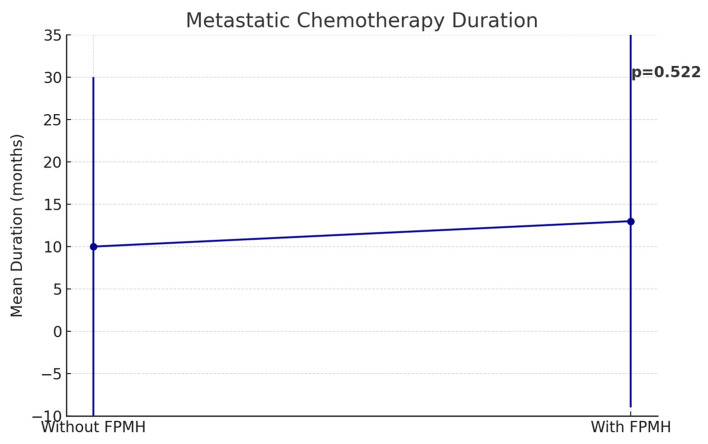
A line graph with error bars representing the mean and standard deviation for metastatic chemotherapy duration, also with its *p*-value displayed.

As shown in Figure 3, chemotherapy-related adverse effects were significantly more frequent in the FPMH group (*p* = 0.036), although other treatment parameters were comparable. While Figure 4 presents the duration of metastatic chemotherapy, indicating no significant difference between groups (*p* = 0.522).

According to Table 3, a significantly higher proportion of patients with FPMH experienced chemotherapy-related adverse effects compared to those without FPMH (50.0% vs. 28.4%, *p* = 0.036). However, other treatment variables and metastatic status showed no significant differences between the groups.

### 3.4. Laboratory Findings and Medication Use

Laboratory parameters, including liver function tests, hematologic indices, and carcinoembryonic antigen (CEA), were comparable between groups. Use of proton pump inhibitors (PPIs) was also similar (17.6% vs. 20.6%, *p* = 1.000) [18].

As shown in Table 4, there were no statistically significant differences in laboratory parameters or proton pump inhibitor (PPI) use between patients with and without a family or personal medical history (FPMH).

**Figure 5:**
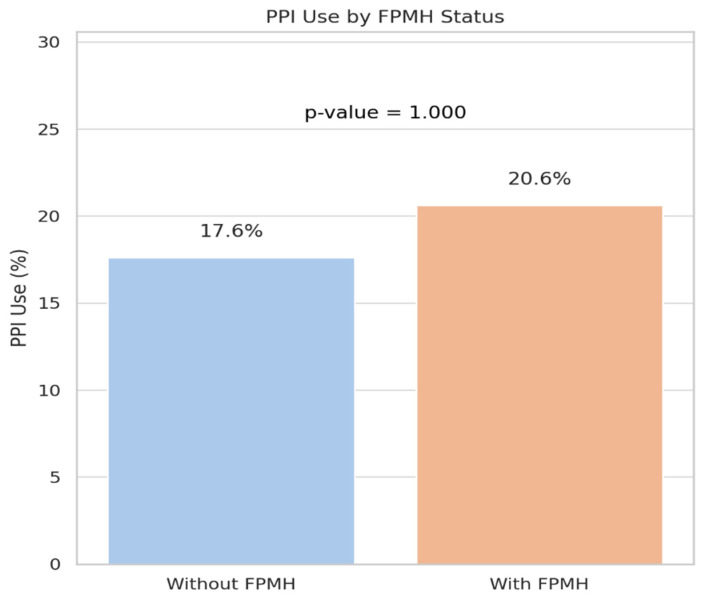
Comparison of PPI use between patients with and without FPMH.

Figure 5 shows that PPI usage was similar across both groups (*p* = 1.000), consistent with the non-significant findings regarding laboratory parameters.

#### Correlation and Regression Analyses

Spearman correlation showed moderate to strong associations between FPMH and several variables: chronic disease (r = 0.423, *p* < 0.01), ECOG status (r = 0.201, *p* < 0.05), recurrence (r = 0.204, *p* < 0.05), and chemotherapy toxicity (r = 0.197, *p* < 0.05).

Univariate logistic regression revealed that FPMH increased the odds of the following:

Having a chronic disease (OR = 7.68, *p* < 0.001).

Recurrence (OR = 2.70, *p* = 0.020).

Chemotherapy-related toxicity (OR = 2.52, *p* = 0.023).

ECOG ≥ 3 had an odds ratio of 9.77 (*p* = 0.052), indicating a borderline association.

Importantly, no significant associations were observed between FPMH and survival duration or genetic mutation status, despite significant differences in recurrence and toxicity, suggesting that FPMH may influence treatment tolerance and post-treatment outcomes more than inherent tumor biology.

**Figure 6:**
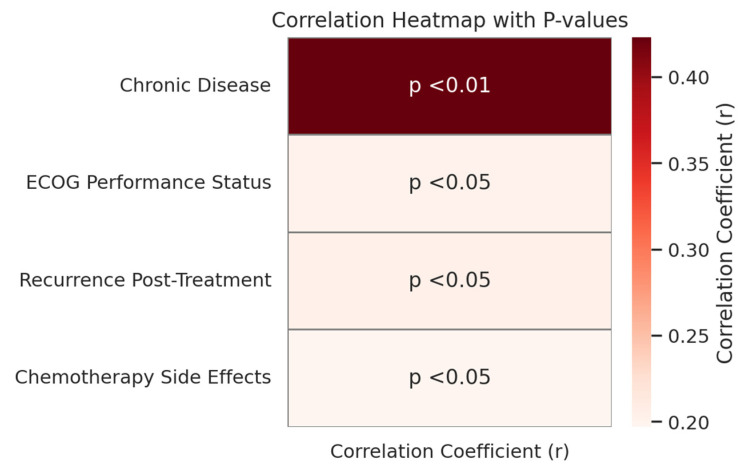
Strength of associations between clinical variables and outcome, with significance levels.

As visualized in Figure 6, moderate to strong correlations were observed between FPMH, chronic disease, ECOG performance, recurrence, and chemotherapy toxicity.

As detailed in Table 5, FPMH status showed a moderate positive correlation with the presence of chronic disease (r = 0.423, *p* < 0.01), and weak but significant correlations with ECOG performance status, recurrence post-treatment, and chemotherapy side effects (*p* < 0.05 for each).

### 3.5. Survival Analysis

Kaplan–Meier survival analysis showed no significant difference in overall survival (OS) between the FPMH and non-FPMH groups (log-rank *p* = 0.73), confirming initial observations. Median OS was 28.7 months for FPMH patients vs. 29.1 months for non-FPMH.

Similarly, progression-free survival (PFS) did not differ significantly (log-rank *p* = 0.61), with median PFS at 10.2 months (FPMH) vs. 11.5 months (non-FPMH).

### 3.6. Cox Regression Analysis

Univariate Cox regression identified the following:

ECOG ≥ 2 was associated with significantly shorter OS (HR = 2.38; 95% CI: 1.22–4.63; *p* = 0.011).

Advanced stage (III–IV) also predicted worse OS (HR = 1.79; 95% CI: 1.02–3.11; *p* = 0.042).

FPMH was not a statistically significant predictor of OS (HR = 1.08; 95% CI: 0.65–1.80; *p* = 0.761).

### 3.7. Predictors of FPMH (Logistic Regression)

To identify predictors associated with the presence of FPMH, we performed multivariate logistic regression:

Chronic disease strongly predicted FPMH (OR = 5.91; 95% CI: 2.38–14.65; *p* < 0.001).

ECOG ≥ 2 was marginally associated with FPMH (OR = 2.13; *p* = 0.068).

**Figure 7:**
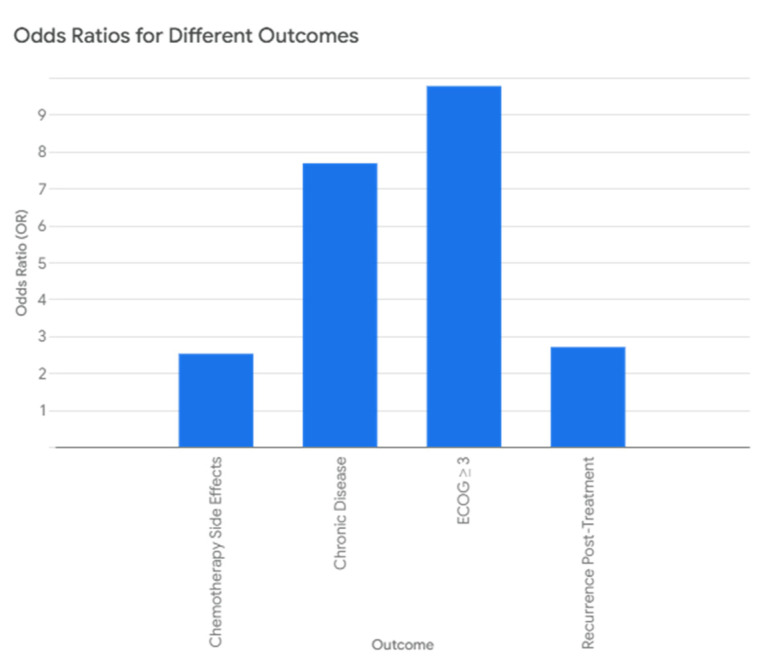
Observed odds ratios for specific conditions.

Mutation status (EGFR/ALK/ROS1), recurrence, or sex did not independently predict FPMH. Figure 7 further illustrates that FPMH significantly increased the odds of chronic disease, post-treatment recurrence, and chemotherapy-related side effects.

As shown in Table 6, logistic regression analysis revealed that patients with FPMH were significantly more likely to have chronic disease (OR = 7.68, *p* < 0.001), recurrence after treatment (OR = 2.70, *p* = 0.020), and chemotherapy side effects (OR = 2.52, *p* = 0.023). The association with poor ECOG performance (≥3) approached significance (*p* = 0.052).

## 4. Discussion

This study explored the impact of family and personal medical history (FPMH) on clinical and treatment outcomes among NSCLC patients treated with tyrosine kinase inhibitors (TKIs). Several key associations were identified, including a significantly higher prevalence of chronic diseases, poorer ECOG performance status, greater recurrence rates, and increased chemotherapy toxicity in patients with FPMH. However, no significant differences were observed in survival outcomes or in the prevalence of oncogenic mutations (e.g., EGFR, ALK, ROS1) [19].

The elevated chronic disease burden in FPMH patients likely reflects shared genetic or environmental risk factors, and may contribute to functional impairment, as evidenced by higher ECOG scores. Worse performance status in turn could affect treatment tolerance and increase the likelihood of recurrence and toxicity. Yet, the lack of differences in overall survival suggests that while FPMH may influence quality of life and treatment complications, it does not appear to affect the fundamental trajectory of the disease under TKI therapy [20,21].

Interestingly, the similarity in survival and mutation status between groups implies that the underlying tumor biology—including molecular drivers like EGFR or ALK—is not significantly impacted by FPMH in this cohort. One possible explanation is that TKI therapy, which targets specific mutations, remains effective regardless of comorbidity status or inherited cancer predisposition. Alternatively, it is possible that the small sample size of the FPMH group limited statistical power to detect subtle differences.

Previous studies have shown mixed results regarding the influence of family history on treatment outcomes in lung cancer. Some suggest that inherited predispositions may increase mutation prevalence or influence prognosis, while others find no survival impact. Our findings align with the latter, indicating that while FPMH may shape the patient’s overall health profile, it may not significantly alter the tumor’s genetic behavior or TKI responsiveness [22,23].

One possible biological link between FPMH and treatment outcomes could be pharmacogenomic variability—for example, inherited differences in drug metabolism enzymes, immune response genes, or DNA repair pathways. These factors might influence chemotherapy tolerance or recurrence risk, but further mechanistic studies are needed to confirm such hypotheses.

This study also has important limitations. Its retrospective design limits causal inference, and FPMH data were extracted from medical records, which may be incomplete or inconsistently documented. Furthermore, the group sizes were imbalanced (34 with FPMH vs. 102 without), which could introduce bias or reduce statistical power. Larger prospective studies with more granular genetic and clinical data are needed to validate these findings and explore underlying mechanisms [24].

## 5. Conclusions

### 5.1. Summary

This study demonstrates that family and personal medical history (FPMH) is associated with key clinical outcomes in NSCLC patients treated with tyrosine kinase inhibitors (TKIs). Specifically, FPMH was linked to a higher prevalence of chronic diseases, poorer ECOG performance status, increased post-treatment recurrence, and greater chemotherapy toxicity. However, no significant differences were observed in overall survival or tumor mutation profiles, suggesting that FPMH may influence the treatment experience rather than the biological behavior of the tumor.

These findings highlight the potential value of considering FPMH in risk stratification and clinical decision-making. Patients with FPMH may benefit from closer monitoring, supportive care measures, and proactive toxicity management.

### 5.2. Recommendations

Given the retrospective nature of this study and the small sample size in the FPMH subgroup, these associations should be interpreted with caution. Prospective studies with larger, balanced cohorts and integrated genomic data are needed to validate these observations and explore the biological mechanisms underlying FPMH-associated differences.

Incorporating FPMH into clinical assessment models may enhance personalized care in NSCLC, especially in predicting treatment-related complications. Further research should focus on defining specific FPMH elements (e.g., type of chronic disease, familial cancer patterns) that most strongly predict treatment outcomes.

## Figures and Tables

**Table 1:** Demographic and clinical characteristics of NSCLC patients by FPMH status.

Characteristic	Without FPMH (n = 102)	With FPMH (n = 34)	*p*-Value
Gender (Female, %)	54.9 (56/102)	47.1 (16/34)	0.552
Smoking Status (Never, %)	62.7 (64/102)	55.9 (19/34)	0.612
Chronic Disease (Yes, %)	15.7 (16/102)	58.8 (20/34)	<0.001
ECOG ≥ 3 (Yes, %)	1.0 (1/102)	8.8 (3/34)	0.048
Recurrence Post-Treatment (%)	20.6 (21/102)	41.2 (14/34)	0.031
Age (Mean ± SD, years)	62.73 ± 12.62	63.21 ± 12.01	0.846
Smoking Pack-Years (Mean ± SD)	33.03 ± 21.11	39.06 ± 22.6	0.352
Overall Survival (Mean ± SD, months)	29.05 ± 24.03	30.03 ± 29.39	0.846
Recurrence Duration (Mean ± SD, months)	44.79 ± 36.85	51.94 ± 43.49	0.363

**Table 2:** Tumor characteristics, staging, and genetic biomarkers by FPMH status.

Characteristic	Without FPMH (n = 102)	With FPMH (n = 34)	*p*-Value
TNM Stage 1–2 (%)	17.6 (18/102)	23.5 (8/34)	0.720
EGFR Mutation (Yes, %)	83.3 (85/102)	91.2 (31/34)	0.402
ALK Mutation (Yes, %)	12.7 (13/102)	2.9 (1/34)	0.192
ROS1 Mutation (Yes, %)	2.9 (3/102)	5.9 (2/34)	0.792
Surgical Intervention (Yes, %)	16.7 (17/102)	29.4 (10/34)	0.172
Non-Squamous Histology (%)	97.1 (99/102)	94.1 (32/34)	0.792
PD-L1 Expression (Mean ± SD, %)	2.35 ± 11.07	0.76 ± 4.29	0.562

**Table 3:** Treatment processes and metastatic status by FPMH status.

Characteristic	Without FPMH (n = 102)	With FPMH (n = 34)	*p*-Value
Neoadjuvant Therapy (Yes, %)	4.9 (5/102)	5.9 (2/34)	1.000
Adjuvant Chemotherapy (Yes, %)	9.8 (10/102)	14.7 (5/34)	0.635
Metastatic Disease (Yes, %)	90.2 (92/102)	94.1 (32/34)	0.727
Chemotherapy Adverse Effects (%)	28.4 (29/102)	50.0 (17/34)	0.036
First-Line Response (CR, %)	12.7 (13/102)	14.7 (5/34)	0.950
Metastatic Chemo Duration (Mean ± SD, months)	9.82 ± 18.25	13.06 ± 17.65	0.522

**Table 4:** Laboratory parameters and drug use by FPMH status.

Characteristic	Without FPMH (n = 102)	With FPMH (n = 34)	*p*-Value
CEA (Mean ± SD, ng/mL)	80.36 ± 182.7	88.91 ± 238.74	0.782
AST (Mean ± SD, U/L)	23.53 ± 20.79	22.53 ± 12.67	0.909
WBC (Mean ± SD, per μL)	8327.42 ± 3707.12	8365.67 ± 3175.8	0.959
Hemoglobin (Mean ± SD, g/dL)	12.21 ± 1.8	12.46 ± 1.88	0.517
PPI Use (Yes, %)	17.6 (18/102)	20.6 (7/34)	1.000

**Table 5:** Correlation analysis of FPMH with clinical variables.

Variable	Correlation Coefficient (r)	*p*-Value
Chronic Disease	0.423	<0.01
ECOG Performance Status	0.201	<0.05
Recurrence Post-Treatment	0.204	<0.05
Chemotherapy Side Effects	0.197	<0.05

**Table 6:** Logistic regression analysis of FPMH effects.

Outcome	Odds Ratio (OR)	*p*-Value
Chronic Disease	7.68	<0.001
ECOG ≥ 3	9.77	0.052
Recurrence Post-Treatment	2.70	0.020
Chemotherapy Side Effects	2.52	0.023

## Data Availability

No new data were created or analyzed in this study.
